# Cosmological flux noise and measured noise power spectra in SQUIDs

**DOI:** 10.1038/srep28275

**Published:** 2016-06-20

**Authors:** Christian Beck

**Affiliations:** 1 School of Mathematical Sciences, Queen Mary University of London, Mile End Road, London E1 4NS, UK

## Abstract

The understanding of the origin of 1/*f* magnetic flux noise commonly observed in superconducting devices such as SQUIDs and qubits is still a major unsolved puzzle. Here we discuss the possibility that a significant part of the observed low-frequency flux noise measured in these devices is ultimately seeded by cosmological fluctuations. We consider a theory where a primordial flux noise field left over in unchanged form from an early inflationary or quantum gravity epoch of the universe intrinsically influences the phase difference in SQUIDs and qubits. The perturbation seeds generated by this field can explain in a quantitatively correct way the form and amplitude of measured low-frequency flux noise spectra in SQUID devices if one takes as a source of fluctuations the primordial power spectrum of curvature fluctuations as measured by the Planck collaboration. Our theoretical predictions are in excellent agreement with recent low-frequency flux noise measurements of various experimental groups. Magnetic flux noise, so far mainly considered as a nuisance for electronic devices, may thus contain valuable information about fluctuation spectra in the very early universe.

The origin of 1/*f* noise in superconducting devices such as SQUIDs and qubits has remained an unsolved puzzle over the past 30 years^1^,^2^,^3^,^4^,^5^,^6^,^7^,^8^,^9^,^10^,^11^. This noise limits the coherence time of superconducting qubits. In contrast to other types of noises, it is notoriously difficult to construct a plausible model of 1/*f* noise that is well-motivated on physical grounds. For superconducting devices such as qubits and SQUIDs, very precise measurements of the flux noise spectrum have recently become available, both in the low-frequency region (10^−4^ …10^−1^ Hz)^3^,^4^ as well as in the high frequency region (0.2 …20 MHz)^8^. Still a fully convincing theory of the origin of the noise, in particular in the low-frequency region, has not been achieved so far. Note that ‘noise’ with a frequency of order 10^−4^ Hz as measured in ref. 4 corresponds to a stochastic process that changes on a time scale of hours, which is difficult to realize on an atomic or molecular level.

A useful effective model discussed in refs 4,6,10 is to attribute the 1/*f* flux noise to fluctuating spins of localized surface electrons, assuming a very broad spectrum of local relaxation times. However, the areal density of spins necessary to fit the observed typical magnitude of the flux noise (5 · 10^17^ *m*^−2^) is much higher than one would normally expect for the materials considered^4^. Moreover, recent measurements of Anton *et al*.^6^ cannot be explained with the assumption of independent surface spins, one needs to assume clustered collective behavior of many spins. While some experimental and theoretical progress has been made in the past years on the (very weak) dependence of how the flux noise couples into the measuring device as a function of its shape and other parameters^3^,^4^,^6^,^9^,^10^, the deeper reason for the *a priori* origin of the magnetic flux noise is still not understood, in particular in the low-frequency region *f* < 1 Hz, where it is most intensive.

This has lead to a search for alternative explanations of the flux noise, pointing towards other candidate sources in different areas of science. For example, a recent attempt of Wang *et al*.^11^ relates some of the flux noise to absorbed oxygen molecules on the surface of the SQUID. If this is true, then removing oxygen adsorbates from the surface of SQUIDs would substantially reduce the flux noise amplitude, a fact that could be experimentally tested in the future. It is likely that the ultimate theory of magnetic flux noise in SQUIDs will point to a combination of many effects, some of them more fundamental than others.

In this paper we propose a new seed mechanism for the generation of flux noise in SQUIDs at a fundamental level. Our theory is in excellent agreement with experimental observations and goes to a much deeper level of what the ultimate source of the flux noise is, and why it is hard to shield and avoid this noise at all. We propose that a significant part of the flux noise at low frequencies is produced by cosmological seeds. We will relate the intrinsic source to the power spectrum of primordial density fluctuations in the early universe^12^,^13^,^14^, conserved to the current time by a suitable cosmological field whose properties will be described in detail. The primordial fluctuations are usually assumed to have been generated by quantum fluctuations of the inflaton field during cosmological inflation. They can be conserved to the current time in terms of misalignment angle fluctuations of a very light frozen-in field that is a relict of the inflationary or quantum gravity phase of the universe.

We will show that misalignment fluctuations can create flux fluctuations. When the Earth moves through the cosmologically generated pattern of small perturbations of the misalignment angle, mirror fluctuations are induced for the phase difference of the measuring Josephson junction. The experimental consequence is 1/*f* flux noise, which, as we will show, has the correct order of magnitude to explain the observed experimental data in the low-frequency region^3^,^4^,^5^. Surface effects, e.g. localized electrons or oxygen molecules^11^, are not in contradiction to this theory, rather, they further modify the cosmological seed signal at higher frequencies (*f* > 1 Hz). In the low frequency region (*f* ≤ 1 Hz), we obtain excellent quantitative agreement with experimentally measured flux noise spectra without fitting any parameter.

Our proposed explanation of the flux noise falls into the general category of experiments that test for tiny measurable fluctuations generated by the Earth moving relative to a given cosmic background field (see e.g. ref. 15 for another recent suggestion based on laser interferometry and a movement of the Earth relative to the cosmic microwave background). If successful, these types of measurements could open up a new experimental ‘window’ to obtain information on the state of the universe at earliest times.

## Results

### Theoretical prediction of a cosmological flux noise power spectrum

The theory developed in this paper gives a concrete prediction for the primary flux noise power spectrum generated in a SQUID due to cosmological fluctuations:


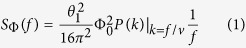


Here *P*(*k*) is the primordial power spectrum of cosmological density fluctuations, as generated e.g. in inflationary models, and *v* is the velocity of the Earth relative to the cosmic field background. Φ_0_ = *h/*2*e* denotes the flux quantum. The angle *θ*_1_ ∈ [−*π, π*] denotes the initial value of the misalignment angle of the frozen-in cosmological field that conserves the power spectrum to the current time. Taking for *P*(*k*) the primordial power spectrum of scalar perturbations that is measured by the Planck satellite^12^,^13^,


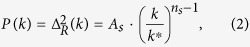


where *A*_*s*_ = (2.14 ± 0.05) ⋅ 10^−9^, *n*_*s*_ = 0.968 ± 0.006, and k^*^ = 0.05 Mpc^−1^, we get the concrete prediction


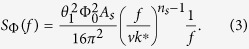


This generates flux noise with an 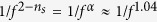 power spectrum. For the squared amplitude of this noise we obtain at *f* = 1 Hz, assuming *v* ≈ 368 km/s (the velocity of the Earth relative to the reference frame set by the cosmic microwave background)


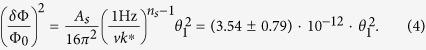


The error bars for the above numerical prediction are dominated by the precision by which the exponent *n*_*s*_ is known (we used the value *n*_*s*_ = 0.968 ± 0.006 provided by the Planck collaboration in ref. 13). The dependence on the velocity *v* in the above formula is very weak because *n*_*s*_ is close to 1. Hence uncertainties in the knowledge of *v* induce only minor numerical differences. For example, changing the velocity *v* ≈ 0.001*c* → *c* by a factor 1000, the amplitude of the predicted flux noise increases just by 11%.

There is no *a priori* way to predict the initial value *θ*_1_ of the cosmological field angle, which arises due to spontaneous symmetry breaking at the Planck scale. Still its order of magnitude can be estimated by assuming that every value of *θ*_1_ ∈ [−*π*, *π*] is equally likely. By taking the uniform average, one obtains the average squared value


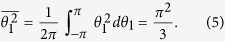


Putting this into eq. (4) one obtains the concrete numerical prediction


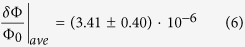


at *f* = 1 Hz. Once again let us mention that this is our prediction of the *primary* flux noise power spectrum in SQUIDs as generated by cosmological effects. This is then further modified by non-universal effects in a given physical realization of a SQUID, which depend (weakly) on dimensions of the SQUID and material parameters, in particular in the high-frequency region *f* > 1 Hz. On the other hand, in the low frequency region *f* ≤ 1 Hz, if a suitable experiment sensitive to these low frequencies is performed, then the measured magnetic flux noise spectrum in the SQUID is expected to be close to the primary form as generated by cosmological seeds.

### Comparison with experimental data

**Figure 1:**
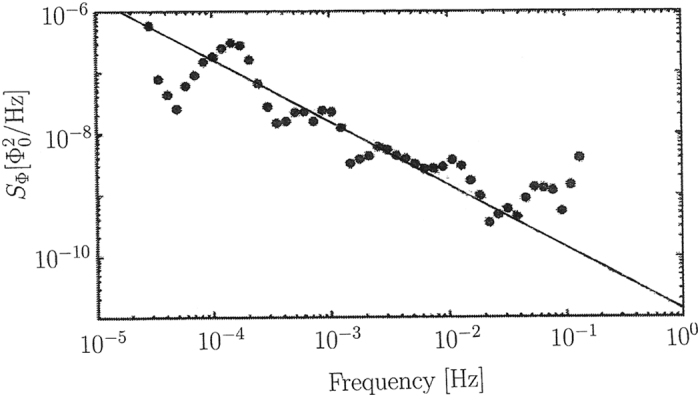
Low-frequency flux noise power spectrum as measured by Bialczak*et al.*^4^, and comparison with the theoretical prediction eq. (3) with 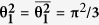 and *α* = 2 − *n*_*s*_ ≈ 1.04 (straight line).

Our predicted noise strength (6) as well as the entire form of the spectrum is in excellent agreement with experimental results. Let us first discuss the seminal flux noise measurements of Bialczak *et al*.^4^ that for the first time reached the low-frequency region 10^−5^ Hz < *f* < 10^−1^ Hz. Figure 1 shows these data together with our theoretical prediction given by eq. (3), using for 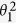 the cosmological average value 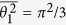. Excellent agreement is found. Note that no parameters are fitted, the theoretical prediction is just as it is, and it agrees perfectly with the data.

**Figure 2:**
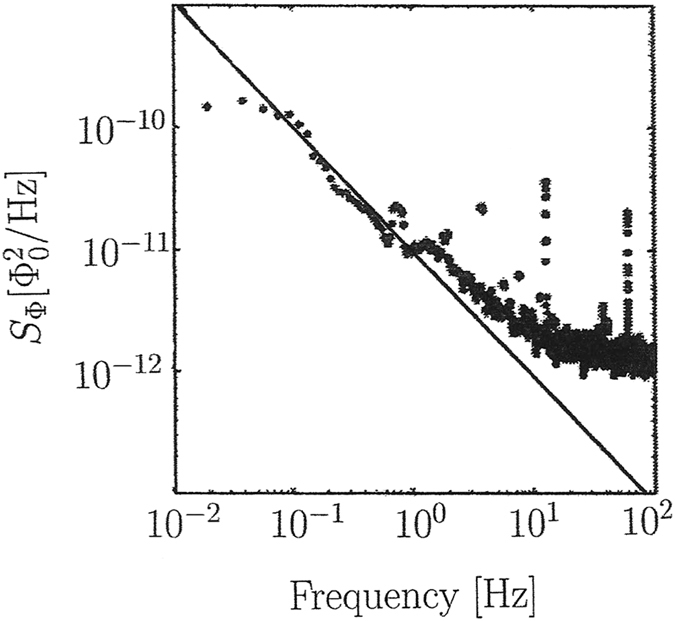
Flux noise power spectrum in the region 0.01…100 Hz as measured by Sendelbach *et al.*^5^ and comparison with the theoretical prediction eq. (3) with 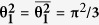 and *α* = 2 − *n*_*s*_ ≈ 1.04 (straight line).

The above measurements did not cover frequencies larger than 10^−1^ Hz. In another experiment conducted by Sendelbach *et al*.^5^, a higher frequency region was probed, these data are displayed in Fig. 2. Again our theoretical prediction (3) agrees very well with the data in the low-frequency region 10^−1^ …10^−0^ Hz. For frequencies larger than about 1 Hz, it is well-known (and verified in Fig. 2) that the noise spectrum becomes flatter, leading effectively to 1/*f*^ α^ noise with α < 1, see e.g. ref. 6 for recent very detailed measurements in this frequency region. In this region secondary (non-universal) effects such as random flips of impurities in the surface material become important, see ref. 10 for suitable models in this direction. Cosmological flux noise can still trigger these complex internal surface processes, leading e.g. to the formation of clusters of surface spins. However, in its original form the cosmological flux noise is most dominant in the region *f* ≪ 1 Hz, where it can be identified by generating an exponent *α* = 2 − *n*_*s*_ ≈ 1.04 > 1.

**Figure 3:**
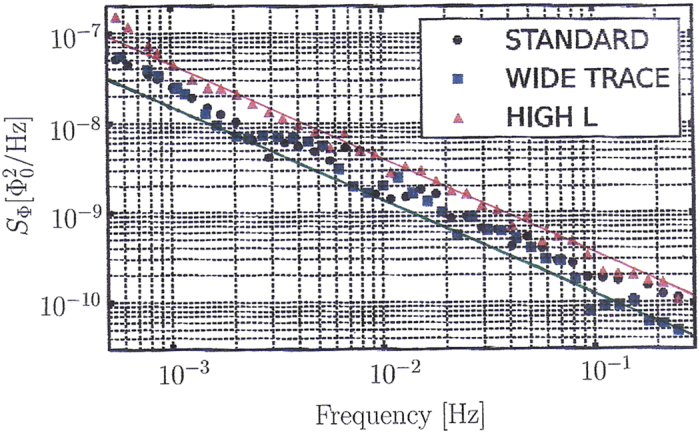
Precision measurements of Sank *et al.*^3^ of the low-frequency flux noise power spectrum and comparison with the theoretical prediction eq. (3). The green line corresponds to the cosmological average value 
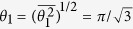
, the red line to the maximum possible value *θ*_1_ = *θ*_max_ = *π*. All experimental data lie between both lines and have the predicted slope 1.04.

Sank *et al*.^3^ have recently performed a new series of high precision flux noise measurements with qubits testing the frequency region *f* = 10^−4^ …10^−1^ Hz. These measurements are the most precise ones currently available. With the new measurement technique described in ref. 3 the fluctuations in the measured noise spectra have become smaller. These recent data are displayed in Fig. 3. Sank *et al*. report a minimum flux noise strength of 3.5 · 10^−6^ if extrapolated to 1 Hz. This minimum value is in very good agreement with our theoretical prediction of flux noise strength as given by eq. (6). The data of Sank *et al*. can be used to estimate the value of the initial misalignment angle *θ*_1_ without any theoretical bias of what it should be. Using eq. (4), we obtain from fitting the standard and wide trace data the value *θ*_1_ = 2.1 ± 0.4.

It is interesting to compare the measured 1/*f*^* α*^ flux noise intensity from various recent experiments and to extract from this the measured value of *θ*_1_. As said before, the pure cosmological 1/*f*^* α*^ flux noise is characterized by an exponent *α* ≈ 1.04, whereas experimental data with an exponent significantly lower than 1 point towards secondary effects, i.e. flux noise generated by surface impurities and other material-dependent effects. Hence, in Table 1 we restricted ourselves to experiments where the exponent *α* was measured to be close to 1 (that is, flux noise data with, say, *α* ≈ 0.6 were ignored whereas data with |*α* − 1.04| < 0.2 did enter our analysis). The result of our analysis in Table 1 is the average value *δ*Φ/Φ_0_ = (3.84 ± 0.96) ⋅ 10^−6^ at 1 Hz, equivalent to *θ*_1_ = 2.04 ± 0.67. Within the error bars, this value is compatible with the cosmological average value 
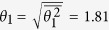
.

## Discussion

Despite intensive research in the past 30 years^1^,^2^,^3^,^4^,^5^,^6^,^7^,^8^,^9^,^10^,^11^ the deeper reason for the occurrence of 1/*f*^* α*^ flux noise in qubits and SQUIDs is still far from being fully understood, in particular in the low-frequency region *f* < 1 Hz. We have shown that cosmologically generated flux noise due to a cosmological field background surrounding the Earth can provide a suitable explanation. The predicted form of the spectrum is in excellent agreement with the recent experimental observations of^3^,^4^,^5^; this agreement is achieved without fitting any parameters. In fact the only relevant parameter involved for the cosmological flux noise is the initial misalignment angle *θ*_1_ ∈ [−*π*, *π*].

As shown in this paper, *θ*_1_ can be extracted from precision measurements of the flux noise intensity. The results of the various experimental groups^3^,^4^,^5^,^6^,^7^,^8^ point to a value *θ*_1_ ≈ 2.04 ± 0.67, compatible with the cosmologically expected average value 1.8. We propose that future systematic experimental tests should aim to separate universal from non-universal (material and device dependent) effects. The universal low-frequency part of the flux noise spectrum may open up a new experimental window to measure power spectra of primordial fluctuations, to provide high-precision measurements of *θ*_1_ and *n*_*s*_, and to ultimately confirm the existence of cosmologically generated flux noise, by systematically excluding other (less fundamental) sources. In fact, these types of experiments could open up a new interdisciplinary field of research which we might call ‘nano-cosmology’.

If the physical interpretation given in this paper is correct, then, rather than being just a nuisance in electronic devices, magnetic flux noise appears to contain valuable information about the state of the universe at an extremely early time, basically looking back to conserved frozen-in quantum fluctuations that were generated at the end of the inflationary period.

## Methods

We will now describe the methods that lead to the theoretical prediction (3) in detail. First, we will show that a spatial scale-invariant spectrum of density perturbations can generate temporal 1/*f* noise for an observer that moves through this fluctuating background with constant velocity. Then we discuss how a primordial power spectrum can be conserved to the current time in terms of misalignment perturbations of a suitable frozen-in cosmological field. As a side product, we show that the potential energy of this frozen-in field can generate constant vacuum energy density that is comparable in magnitude to the currently observed dark energy density in the universe. The coupling of the misalignment fluctuations into Josephson junctions via the flux quantization condition is then discussed in the final subsection.

### 1/*f* noise from a scale-invariant spectrum of spatial density fluctuations

Let us quite generally discuss an environment of energy density *ρ* that exhibits spatial density fluctuations *δρ* described by the (spatial) power spectrum *P*(*k*):


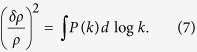


Here we use the definition of power spectrum as used by cosmologists and astrophysicists (which is slightly different from that used by statistical physicists). The astrophysical power spectrum *P*(*k*) as defined in eq. (7) is the variance of the relative density fluctuations *δρ*/*ρ* per logarithmic interval *d* log*k*, where *k* denotes the scale. To evaluate the power spectrum at a particular scale *k*_0_, by convention the borders of the integral in eq. (7) are chosen as *k*_0_ and *ek*_0_. An equivalent way of writing eq. (7) is thus


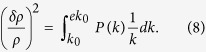


The famous Harizon-Zeldovich spectrum is given by


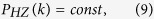


i.e. it is scale invariant. The primordial power spectrum of curvature fluctuations as measured by the Planck satellite is^12^,^13^


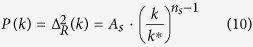


with *A*_*s*_ ≈ 2.2 ⋅ 10^−9^, *n*_*s*_ ≈ 0.96.

Let us now consider an observer that moves with constant velocity *v* through this environment and let 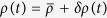 be the local density surrounding the observer at time *t*. Let us consider the dimensionless stochastic process *Y*(*t*) given by 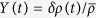, where 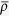 denotes the average density. From eq. (8) it follows that the stochastic process *Y*(*t*) has the temporal power spectrum


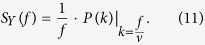


Here we use the definition of (temporal) power spectrum as used by statistical physicists (which has the dimension of time), and *f* denotes the frequency.

From eq. (11) one sees that a medium with a spatial Harrison-Zeldovich spectrum *P*(*k*) of density fluctuations generates a temporal 1/*f* noise for an observer that moves through it with constant velocity. More generally, spatial fluctuations with spectral index *n*_*s*_ generate temporal noise with a power-law spectrum of type 1/*f*^* α*^, where *α* = 2 − *n*_*s*_. Note that often one needs very strong assumptions (such as a uniform distribution of relaxation times^10^) to construct a plausible temporal model for the origin of 1/*f* noise. Here we see that spatial density fluctuations that are nearly-scale invariant provide a very natural way to generate near- 1/*f* noise.

### Conservation of the primordial power spectrum to the current time by a frozen-in cosmological field

Primordial density fluctuations *δρ*/*ρ* are imprinted on any light field that is present during cosmological inflation^16^,^17^,^18^,^19^,^20^. By a light field we actually mean a near-massless scalar field with a mass much smaller than that of the inflaton^21^. Assume there is such a light field during inflation which is a relict from an early quantum gravity epoch. We write this field as *a* = *f*_*a*_*θ*, where *f*_*a*_ is a large energy scale, assumed to be of the order of the Planck scale, and *θ* ∈ [−*π*, *π*] is a dimensionless angle variable. We have chosen the symbol *a* for this field since it may for example be an axion-like field^22^,^23^,^24^,^25^,^26^,^27^,^28^,^29^,^30^.

In the simplest case we may just assume a quadratic potential 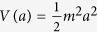, where *m* is the mass of the scalar field under consideration, with *m* ≪ *f*_*a*_. For the QCD axion, a candidate for cold dark matter in the universe, *f*_*a*_~10^11^ GeV, but we are actually thinking here of a different field that is a relict from a quantum gravity epoch, for which *f*_*a*_ is larger, of the order of magnitude of the Planck scale 10^19^ GeV. These types of axion-like fields with large *f*_*a*_ are predicted in a number of quantum gravity and inflationary models^19^,^20^. The scale *f*_*a*_~10^19^ GeV also occurs as a fundamental lattice spacing in a quantum description of discrete geometries where fundamental fields obeying Fermi-Dirac and Bose-Einstein statistics arise in a natural way out of complex quantum network manifolds^31^,^32^.

If the above light field *a* = *f*_*a*_*θ* (which is not the inflaton but an additional light field arising out of a unified theory of quantum gravity) is present during cosmological inflation, then quantum fluctuations during inflation produce spatial fluctuations *δa* of that field given by^27^,^28^


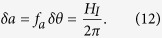


Here *H*_*I*_ is the Hubble parameter during inflation. These field fluctuations correspond to density fluctuations given by


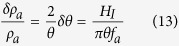


since 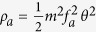 and 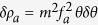. Spatial fluctuations in the energy density of this cosmological field can thus be equivalently regarded as representing (encoding) fluctuations *δθ* of a misalignment angle *θ*, as given by eq. (13). These angle perturbations are present on a huge range of scales, due to the exponential expansion of the scale factor during inflation.

Let us now check the conditions under which these angle perturbations produced during inflation can be conserved to the current time. The equation of motion of the field *a* in an expanding flat Friedmann-Robertson-Walker background is^28^


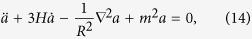


where *H* is the (temperature dependent) Hubble parameter and *R* the scale factor. The spatial gradient terms in the above equation are very small and can be neglected. We want this field to be frozen in up to the current time, in order to conserve the primordial power spectrum, meaning the angle perturbations have not evolved at all so far. This means the kinetic energy 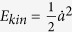 must still be much smaller than the potential energy 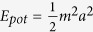. This condition of a frozen-in state is realized if the Hubble damping is still strong enough as compared to the potential strength, i.e. if


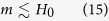


where *H*_0_ is the Hubble parameter at the current time.

On the other hand, as mentioned before, we may assume that this cosmological field is a relict from a quantum gravity epoch, i.e. an epoch where possibly all interactions were in a unified state, and then this symmetry was broken. This requires that the energy scale *f*_*a*_ (which corresponds to a symmetry breaking scale)^22^ should be of the order of the Planck scale, or even higher:


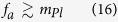


Equations (15) and (16) imply that the mass parameter *m* must be extremely small, 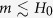, and the energy scale 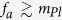 extremely large. Still the product *mf*_*a*_ which enters into the potential energy gives a well-defined finite value which has a physical interpretation, namely we get potential energy that has the same order of magnitude as the currently observed dark energy density *ρ*_*dark*_ in the universe^33^,^34^,^35^:


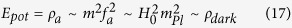


Indeed for a flat universe one has


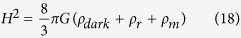


and at the current time (*H* = *H*_0_) the dark energy density *ρ*_*dark*_ is observed to dominate as compared to the radiation density *ρ*_*r*_ and matter density *ρ*_*m*_. Note that in units where 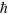 = *c* = 1 we have 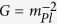.

Hence, as a by-product of our efforts to construct a light field that conserves the primordial power spectrum to the current time, we have obtained dark energy. Dark energy could be identified with the constant potential energy of the frozen-in cosmological field *a*. Since this field is static up to the current time, the energy density does not evolve in time and represents a small cosmological constant.

Note that in contrast to the QCD axion dark matter field, which is initially frozen-in but leaves its frozen-in (time-independent) state shortly before the QCD phase transition to start oscillating behaviour, we are here postulating a different axion-like field which is still in a frozen-in state up to the current time. Its potential energy is not given by QCD vacuum energy (as for the QCD axion) but by the dark energy density *ρ*_*dark*_ in the universe. For the simplest model, a cosmological constant Λ and non-evolving dark energy density, this energy density is given by 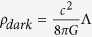. The most recent Planck measurements^13^, based on the Λ*CDM* model, yield the numerical value *ρ*_*dark*_ = (3.35 ± 0.16) GeV/*m*^3^.

In the model proposed in this paper we associate the cosmological field *a* with frozen-in magnetic flux fluctuations associated with a cosmological constant, which are completely decoupled from the rest of the universe, and which do not evolve in time (more complicated models with an evolving *ρ*_*dark*_ can also be studied but are not subject of this paper). It is interesting to check what typical values of magnetic field strength of this ‘dark’ magnetic field *B*_0_ one formally obtains if one assumes that a fraction *η* of the dark energy density *ρ*_*dark*_ in the universe is actually frozen-in magnetic field energy *ρ*_*B*_. Writing 
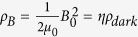
 one obtains


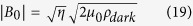


which for *η* = 1 numerically evaluates to |*B*_0_| = (3.67 ± 0.08) ⋅ 10^−8^ T provided one uses for *μ*_0_ the usual magnetic permeability of the vacuum. This is a very small magnetic field, comparable in size to small magnetic fields measured in the outer heliosphere. It is unlikely that the above formal magnetic field *B*_0_ can ever be measured, since it can point into any direction of space equally likely. Still it is interesting to check what typical area A^*^ one obtains if one writes down a flux quantization condition of the form


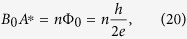


where *n* is an integer. For the choice *n* = 1 = *η* we obtain the numerical value A^*^ = (5.64 ± 0.12) ⋅ 10^−8^ *m*^2^, which corresponds to a length scale 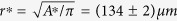. This is of the same order of magnitude as the loop radius of a typical (big) SQUID. It is encouraging that one does not get any exotic length scales but parameters that make sense in SQUID physics. While it is unlikely that the above formal magnetic field *B*_0_ associated with dark energy can ever be measured directly, our main proposal in this paper is that tiny fluctuations and inhomogenities of the associated flux can be measured in a highly sensitive SQUID environment, and lead to the experimentally observed flux noise. This will be worked out in more detail the following section.

### Coupling of misalignment angle fluctuations into SQUIDs and qubits

While the potential energy of the field *a* is practically constant, and the field is very homogeneous, there are still tiny spatial density fluctuations imprinted onto this nearly massless field, originating from quantum fluctuations during inflation. These fluctuations of the field *a* are equivalent to tiny conserved spatial misalignment angle fluctuations, and they should still have the same power spectrum as in the very early universe.

Let us now discuss a possible mechanism how a fluctuation of the misalignent angle surrounding locally the moving Earth can couple into Josephson junctions, SQUIDs or qubits. Let us first consider standard SQUID physics. If two Josephson junctions, one described by the gauge-invariant phase difference *φ*_1_ and the other one by the gauge-invariant phase difference *φ*_2_ form a SQUID (superconducting quantum interference device), then it is well-known that the difference *φ*_1_ − *φ*_2_ satisfies^36^


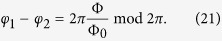


Here Φ is the magnetic flux included in a closed loop containing the weak link region of the SQUID, and Φ_0_ = *h*/2*e* denotes the flux quantum. Eq. (21) is a simple consequence of the fact that the joint macroscopic wave function describing the physics of both junctions forming the SQUID must be unique^36^.

From the above it is obvious that an uncertainty *δφ* in the phase difference *φ*_1_ − *φ*_2_ can be equivalently regarded as a flux uncertainty *δ*Φ:


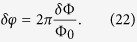


Fluctuations in (or uncertainties in the knowledge of) the angle variable of a Josephson junction thus imply magnetic flux noise.

In analogy to this, in ref. 29 it was proposed that phase differences in Josephson junctions are influenced by phase differences of a surrounding axion condensate, in the sense that any change *δθ* in the surrounding axion condensate is accompanied (or compensated) by a mirror change *δφ* of the electromagnetic phase difference in the corresponding Josephson junction,


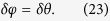


The physical meaning of eq. (23) is that the axion field sets the background to which all Josephson phases need to be related. If the background changes, so does the Josephson phase. As the Earth moves through a spatially inhomogeneous axion background, the axion misalignment angle exhibits tiny changes *δθ* which are accompanied by a corresponding mirror change of the electromagnetic phase variable in the junction. From eq. (23) we get


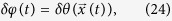


where 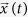 is the position of the Josephson junction on the Earth moving relative to the background field of spatial misaligment angle fluctuations.

While for a more detailed discussion of the underlying mathematics we refer to^29^,^30^, let us here give a simple physical argument why a SQUID-like interaction of the form (23) is the only consistent way to introduce a coupling between axion fields and Josephson junctions. Axions are described by a cosine potential 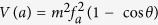 in the angle variable *θ* = *a*/*f*_*a*_, and the physical effect of any perturbation *δθ* of the angle must be invariant under the transformation *δθ* → *δθ* + 2*π*. Moreover, also SQUID physics is invariant under the transformation *δφ* → *δφ* + 2*π*, as only the phase modulo 2*π* of the macroscopic wave function matters. Whatever the interaction between SQUIDs and axions, performing both transformations simultaneously should not change the physics. If we assume a linearized relation of the form


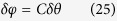


with some unknown coupling constant *C*, then any physics should be invariant under the above transformations of increasing the angle perturbations by 2*π* on either side. Hence


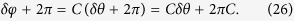


Since *δφ* = *Cδθ* we thus obtain


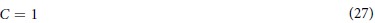


which proves eq. (23).

Combining eqs (13), (22) and (24), we get a concrete prediction for the flux noise generated by the background misalignment fluctuations:


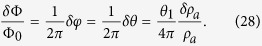


Here *θ*_1_ ∈ [−*π*, *π*] denotes the initial value of the cosmological field angle. Using also eq. (11) we end up with


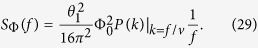


In particular, the primordial power spectrum of scalar perturbations (10) yields the prediction


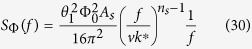


which is the main result of this paper.

Our derivation of eq. (30) was based on the assumption of a simple quadratic potential *V*(*a*) for the cosmological field *a*. More general, for a given arbitrary potential *V*(*a*) one obtains the more general result that the effective coupling parameter *θ*_1_ in eq. (29) and (30) is given by


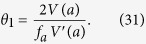


Here *a* = *a*(0) = *f*_*a*_*θ*(0) denotes the initial field value of the cosmological field *a*. For example, for a cosine potential 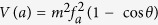 one obtains


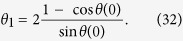



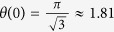
 implies *θ*_1_ = 2.55, still compatible with the flux noise measurements in Table 1.

## Additional Information

**How to cite this article**: Beck, C. Cosmological flux noise and measured noise power spectra in SQUIDs. *Sci. Rep.* **6**, 28275; doi: 10.1038/srep28275 (2016).

## Figures and Tables

**Table 1:** Flux noise strength at frequency 1 Hz as measured in different recent experiments^3^,^4^,^5^,^6^,^7^,^8^.

Experiment	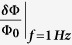	Remarks
Sank *et al*.^4^	3.9 ⋅ 10^−6^	standard and wide trace
Sank *et al*.^4^	5.5 ⋅ 10^−6^	high L, extrapolated to 1 Hz
Sendelbach *et al*.^5^	3.5 ⋅ 10^−6^	direct measurement at 1 Hz
Bialczak *et al*.^3^	4.0 ⋅ 10^−6^	extrapolated to 1 Hz
Anton *et al*.^7^	3.5 ⋅ 10^−6^	direct measurement at 1 Hz
Anton *et al*.^6^	4.4 ⋅ 10^−6^	direct measurement at 1 Hz
Bylander *et al*.^8^	2.1 ⋅ 10^−6^	extrapolated from 1 MHz to 1 Hz
average	(3.84 ± 0.96) ⋅ 10^−6^	sample condition |*α* − 1.04| < 0.2
